# Features of the brain proteome of mice with a model of affective disorders

**DOI:** 10.1192/j.eurpsy.2026.11275

**Published:** 2026-07-31

**Authors:** T. Amstislavskaya, K. Smirnova, E. Dmirtieva, M. Zavialova, L. Smirnova

**Affiliations:** 1 Scientific Research Institute of Neurosciences and Medicine, Novosibirsk; 2 Laboratory of Molecular Genetics and Biochemistry, Research Institute of Mental Healthomsk National Research Medical, Tomsk; 3 Skolkovo Institute of Science and Technology, Moscow, Russian Federation

## Abstract

**Introduction:**

The key role of the DISC1 protein in the nervous system is the regulation of neurogenesis. Even minor changes in the DISC1 protein structure lead to an increased risk of developing mental disorders. To study the mechanisms underlying the development of psychopathologies, a mouse model with a mutation in the DISC1 gene was created. Disc1-Q31L-/- (Q31L) mice with the mutation are a recognized model of affective disorders.

**Objectives:**

To study the features of the proteomic composition of the cortex, hippocampus and hypothalamus in a Q31L mouse.

**Methods:**

Were used male 20 mice from two genetic strains: C57BL/6 (hereinafter WT) and homozygous Disc1-Q31L-/- (Q31L) mice. The animals were obtained from the “Biological Collection – genetic biomodels of neuropsychiatric diseases” by Scientific Research Institute of Neuroscience and Medicine in Novosibirsk.Mice were sacrificed by decapitation and brain structures were collected and immediately frozen. Brain samples for mass spectrometry were prepared using a standard protocol for S-trep filters. After trypsinolysis, proteins were identified using HPLC/mass spectrometry on an Orbitrap instrument. Mass spectrometric analysis was performed on the Advanced Mass Spectrometry Core Facility of Skolkovo Institute of Science and Technology.

**Results:**

Compared to WT mice, 295 unique proteins were identified in the cerebral cortex of Q31L mice. Among these, the most abundant were proteins regulating translation and transcription processes in mitochondria and ribosomes, mRNA splicing, and phosphorylation processes. In the hippocampus of Q31L mice, 401 unique proteins were identified compared to WT mice. Also highly abundant were proteins regulating translation, transcription, transfection, and ubiquitination processes, and of particular interest were proteins of the trafficking of AMPA receptors, and рostsynaptic density protein 95 clustering complex. In the hypothalamus of mice the maximum number of unique proteins (447) was identified, also primarily represented by ribosomal and mitochondrial proteins regulating protein synthesis.

**Conclusions:**

A number of unique proteins have been identified that characterize the brains of Q31L mice. There is every reason to believe that these proteins are involved in the pathogenesis of affective disorders, but the mechanisms they regulate remain to be elucidated.

Support by the Russian Science Foundation grant No. 23-75-00023

**Disclosure of Interest:**

None Declared

